# Supplementary material for: Loads of Coliforms and Fecal Coliforms and Characterization of Thermotolerant Escherichia coli in Fresh Raw Milk Cheese
Source: Foods. 2022 Jan 25;11(3):332. doi: 10.3390/foods11030332 (PMC8834472; doi:10.3390/foods11030332)
Supplement: Supplementary file 1 [file foods-11-00332-s001.zip › foods-1543838-supplementary.pdf]

Supplementary Table S1. Primers used for PCR

Primer	Target	Sequence 5'--3'	Reference
**1- β-Lactamases**			
TEM-F	*bla*$_{TEM}$	ATAAAATTCTTGAAGACGAAA	[1]
TEM-R		GACAGTTACCAATGCTTAATC	
SHV-F	*bla*$_{SHV}$	TTATCTCCCTGTTAGCCACC	[1]
SHV-R		GATTTGCTGATTTCGCTCGG	
OXA- F	*bla*$_{OXA}$	TCAACTTTCAAGATCGCA	[2]
OXA-R		GTGTGTTTAGAATGGTGA	
CTX-M-F	*bla*$_{CTX-M}$	CGCTTTGCGATGTGCAG	[3]
CTX-M-R		ACCGCGATATCGTTGGT	
CMY-F	*bla*$_{CMY}$	GACAGCCTCTTTCTCCACA	[4]
CMY-R		TGGAACGAAGGCTACGTA	
OXY-F	*bla*$_{OXY}$	GTTTTGGTAACTGTGACGGG	[5]
OXY-R		AGAGTGCAGAGTGTTGCAG	
**2- Plasmid-mediated quinolone resistance genes**			
qnrA-F	*qnrA*	ATTTCTCACGCCAGGATTTG	[6]
qnrA-R		GATCGGCAAAGGTTAGGTCA	
qnrB-F	*qnrrB*	GATCGTGAAAGCCAGAAAGG	[6]
qnrB-R		ACGATGCCTGGTAGTTGTCC	
qnrS-F	*qnrS*	ACGACATTCGTCAACTGCAA	[6]
qnrS-R		TAAATTGGCACCCTGTAGGC	
aac(6')-Ib-F	*aac(6')-Ib*	TTGCGATGCTCTATGAGTGGCTA	[7]
aac(6')-Ib-R		CTCGAATGCCTGGCGTGTTT	
**3- Tetracyclines**			
*tet(*A)-F	*tet*(A)	GCTACATCCTGCTTGCCTTC	[8]
tet(A)-R		CATAGATCGCCGTGAAGAGG	
tet(B)-F	*tet*(B)	TTGGTTAGGGGCAAGTTTTG	[8]
tet(B)-R		GTAATGGGCCAATAACACCG	
tet(C)-F	*tet*(C)	CTTGAGAGCCTTCAACCCAG	[8]
tet(C)-R		ATGGTCGTCATCTACCTGCC	
tet(D)-F	*tet*(D)	AAACCATTACGGCATTCTGC	[8]
tet(D)-R		GACCGGATACACCATCCATC	
tet(E)-F	*tet*(E)	AAACCACATCCTCCATACGC	[8]
tet(E)-R		AAATAGGCCACAACCGTCAG	
**4- Phylogenetic group**			
ChuA.1b	*chuA*	ATGGTACCGGACGAACCAAC	[9]
ChuA.2.2		TGCCGCCAGTACCAAAGACA	
YjaA.1b	*yjaA*	CAAACGTGAAGTGTCAGGAG	[9]
YjaA.2b		AATGCGTTCCTCAACCTGTG	
TspE4C2.1b	*tspE4C*	CACTATTCGTAAGGTCATCC	[9]
TspE4C2b		AGTTTATCGCTGCGGGTCGC	
AceK.f	*arpA*	AACGCTATTCGCCAGCTTGC	[9]
ArpA1.r		TCTCCCCATACCGTACGCTA	
**5- Virulence genes**			
ETEC	*lt*	GGCGACAGATTATACCGTGC	[10]
		CGGTCTCTATATTCCCTGTT	
	st	ATTTTTCTTTCTGTATTGTCTT	[10]
		CACCCGGTACAAGCAGGATT	
EPEC	*bfp*A	AATGGTGCTTGCGCTTGCTGC	[10]
		GCCGCTTTATCCAACCTGGTA	
	*eae*A	GACCCGGCACAAGCATAAGC	[10]
		CCACCTGCAGCAACAAGAGG	
STEC	*stx*1	CTGGATTTAATGTCGCATAGTG	[10]
		AGAACGCCCACTGAGATCATC	
	stx2	GGCACTGTCTGAAACTGCTCC	[10]
		TCGCCAGTTATCTGACATTCTG	
EIEC	*ial*	GGTATGATGATGATGAGTCCA	[10]
		GGAGGCCAACAATTATTTCC	
**6- ERIC–PCR**			
ERIC2	ERIC	AAGTAAGTGACTGGGGTGAGCG	[11]

References

1. Weill, F.X.; Demartin, M.; Tande, D.; Espie, E.; Rakotoarivony, I.; Grimont, P.A. SHV-12-like extended-spectrum-beta-lactamase-producing strains of *Salmonella enterica* serotypes Babelsberg and Enteritidis isolated in France among infants adopted from Mali. *J. Clin. Microbiol.* **2004**, *42*, 2432–2437, https://doi.org/10.1128/JCM.42.6.2432-2437.2004.
2. Ahmed, A.M.; Motoi, Y.; Sato, M.; Maruyama, A.; Watanabe, H.; Fukumoto, Y.; Shimamoto, T. Zoo animals as reservoirs of gram-negative bacteria harboring integrons and antimicrobial resistance genes. *Appl. Environ. Microbiol.* **2007**, *73*, 6686–6690, https://doi.org/10.1128/AEM.01054-07.
3. Bonnet, R.; Sampaio, J.L.; Labia, R.; De Champs, C.; Sirot, D.; Chanal, C.; Sirot, J. A novel CTX-M β-lactamase (CTX-M-8) in cefotaxime-resistant *Enterobacteriaceae* isolated in Brazil. *Antimicrob. Agents Chemother.* **2000**, *44*, 1936–1942, https://doi.org/10.1128/aac.44.7.1936-1942.2000.
4. Briñas, L.; Zarazaga, M.; Saenz, Y.; Ruiz-Larrea, F.; Torres, C. β-lactamases in ampicillin-resistant *Escherichia coli* isolates from foods, humans, and healthy animals. *Antimicrob. Agents Chemother.* **2002**, *46*, 3156–3163, https://doi.org/10.1128/aac.46.10.3156-3163.2002.
5. Fevre, C.; Jbel, M.; Passet, V.; Weill, F.X.; Grimont, P.A.; Brisse, S. Six groups of the OXY β-lactamase evolved over millions of years in *Klebsiella oxytoca*. *Antimicrob. Agents Chemother.* **2005**, *49*, 3453–3462, https://doi.org/10.1128/AAC.49.8.3453-3462.2005.
6. Cattoir, V.; Poirel, L.; Rotimi, V.; Soussy, C.J.; Nordmann, P. Multiplex PCR for detection of plasmid-mediated quinolone resistance *qnr* genes in ESBL-producing enterobacterial isolates. *J. Antimicrob. Chemother.* **2007**, *60*, 394–397, https://doi.org/10.1093/jac/dkm204.
7. Robicsek, A.; Jacoby, G.A.; Hooper, D.C. The worldwide emergence of plasmid-mediated quinolone resistance. *Lancet Infect. Dis.* **2006**, *6*, 629–640, https://doi.org/10.1016/S1473-3099(06)70599-0.
8. Ng, L.K.; Martin, I.; Alfa, M.; Mulvey, M. Multiplex PCR for the detection of tetracycline resistant genes. *Mol. Cell Probes* **2001**, *15*, 209–215, https://doi.org/10.1006/mcpr.2001.0363.
9. Clermont, O.; Christenson, J.K.; Denamur, E.; Gordon, D.M. The Clermont *Escherichia coli* phylo-typing method revisited: Improvement of specificity and detection of new phylo-groups. *Environ. Microbiol. Rep.* **2013**, *5*, 58–65, https://doi.org/10.1111/1758-2229.12019.
10. López-Saucedo, C.; Cerna, J.F.; Villegas-Sepulveda, N.; Thompson, R.; Velazquez, F.R.; Torres, J.; Tarr, P.I.; Estrada-Garcia, T. Single multiplex polymerase chain reaction to detect diverse loci associated with diarrheagenic *Escherichia coli*. *Emerg. Infect. Dis.* **2003**, *9*, 127–131, https://doi.org/10.3201/eid0901.010507.
11. Versalovic, J.; Koeuth, T.; Lupski, J.R. Distribution of repetitive DNA sequences in eubacteria and application to fingerprinting of bacterial genomes. *Nucleic Acids Res.* **1991**, *19*, 6823–6831, https://doi.org/10.1093/nar/19.24.6823.
